# Cnidaria: fast, reference-free clustering of raw and assembled genome and transcriptome NGS data

**DOI:** 10.1186/s12859-015-0806-7

**Published:** 2015-11-02

**Authors:** Saulo Alves Aflitos, Edouard Severing, Gabino Sanchez-Perez, Sander Peters, Hans de Jong, Dick de Ridder

**Affiliations:** 1 Applied Bioinformatics, Plant Research International, Wageningen, The Netherlands; 2 Bioinformatics Group, Department of Plant Sciences, Wageningen University, Wageningen, The Netherlands; 3 Laboratory of Genetics, Wageningen University, Wageningen, The Netherlands

**Keywords:** Clustering, k-mer, NGS, RNA-seq, Phylogeny, Species identification

## Abstract

**Background:**

Identification of biological specimens is a requirement for a range of applications. Reference-free methods analyse unprocessed sequencing data without relying on prior knowledge, but generally do not scale to arbitrarily large genomes and arbitrarily large phylogenetic distances.

**Results:**

We present Cnidaria, a practical tool for clustering genomic and transcriptomic data with no limitation on genome size or phylogenetic distances. We successfully simultaneously clustered 169 genomic and transcriptomic datasets from 4 kingdoms, achieving 100 % identification accuracy at supra-species level and 78 % accuracy at the species level.

**Conclusion:**

CNIDARIA allows for fast, resource-efficient comparison and identification of both raw and assembled genome and transcriptome data. This can help answer both fundamental (e.g. in phylogeny, ecological diversity analysis) and practical questions (e.g. sequencing quality control, primer design).

**Electronic supplementary material:**

The online version of this article (doi:10.1186/s12859-015-0806-7) contains supplementary material, which is available to authorized users.

## Background

Unequivocal identification of biological specimens is a major requirement for reliable and reproducible (bio)medical research, control of intellectual property by biological patent holders, regulating the flow of biological specimen across national borders, enforcing the Nagoya protocol [1] and verifying the authenticity of claims of the biological source of products by customs authority.

Several methods for species identification have been developed based on DNA analysis, that can be classified as probe-based and nucleotide sequencing based methods. Probe-based technologies include microarrays, PCR probes, DNA fingerprinting and immunoassays involving the hybridization of DNA samples with predetermined sets of probes or primers. Such methods are cheap and allow precise identification, but may fail in cases where target DNA is not precisely matched by the probes or primers. Alternatively, nucleotide sequencing methods have been developed to increase accuracy, flexibility and throughput. These can be separated into complete or targeted approaches. Targeted identification of short and highly variable genomic regions by exome capture, Expressed Sequence Tag (EST), DNA barcoding and ribosomal DNA (rDNA) sequencing has been used for many years. Targeted DNA sequencing can be done iteratively for taxonomic identification at subspecies, accession and cultivar levels. Whole Genome Sequencing (WGS) and RNA-seq using Next Generation Sequencing (NGS) technology, examples of complete sequencing methods, have the highest information content of all methods, although its high cost has prevented it from being adopted massively. However, with the recent reduction of costs and increase in throughput, NGS starts to become more prevalent, making it a feasible alternative method for species identification. This calls for the creation of a new a set of tools to comprehensively analyse the deluge of data.

Methods for species identification based on NGS data can be separated into two main classes: reference-based and reference-free methods (reviewed in [2]). Reference-based methods usually map the sequence reads to the genome of a close relative and infer the phylogeny by aligning the observed polymorphisms. This technology requires quality control (cleaning) of the data, mapping the data to the genomic sequence of a close relative, and detection and comparison of polymorphisms [3]. In contrast, reference-free methods (RFMs) are designed to analyse unprocessed sequencing data without any previous knowledge of its identity. The data can be compared against other datasets of unknown samples, in the case of metagenomics comparing population structures [4–13] or against a panel of known species. In the latter case, it can identify a previously unknown sample, giving it an approximate position relative to the known species.

RFMs can be based on the Discrete Fourier Transform (DFT), compression and *k*-mers. DFT methods, such as in [14], transform nucleotide sequences into frequency statistics and compare these for species classification. Although remarkably fast, these methods are not able to store the differences between the genomes for further enquiry, yielding no insight into sequence composition. Compression based methods calculate the distance between pairs of sequences by analysing the reduction in computer memory usage when both sequences are compressed together [15]. However, compression-based methods are time and resource intensive for large genomes or large datasets.

Given a set of samples *S* = {*s*_1_, *s*_2_, …, *s*_*n*_}, represented either by assembled genome or transcriptome sequences (.fasta files) or by unprocessed sequencing data (.fastq files), *k*-mer based methods split the nucleotide sequences into all constituent substrings of length *k*. The presence/absence or counts of these *k*-mers are then used to calculate a dissimilarity *D*(*s*_*i*_, *s*_*j*_) between each sample pair (*s*_*i*_, *s*_*j*_), which should be minimal for samples with identical sequence composition. Several implementations of *k*-mer based RFMs exists, such as FFP [16], CO-PHYLOG [17], NEXTABP [18], MULTIALIGNFREE [19], KSNP [20] and SPACED WORDS/KMACS [21]. Although their underlying principles are generally useful for the analysis of large data collections, most implementations are designed for either analysis of a limited portion of the data, such as organelles or ribosomal DNA, or analysis of closely related species (such as bacteria, in metagenomics applications). As a consequence, it is not feasible to apply these tools on large amounts of whole-genome sequencing data or to analyse data that spans large phylogenetic distances. Two exceptions are the AAF [22] and REFERENCEFREE [23]. In AAF, the authors successfully clustered infra-family plant species using whole genome sequencing data; in REFERENCEFREE, it was demonstrated that it is possible to find polymorphisms shared by subsets of samples by counting and merging sets of k-mers. This latter method was effectively applied in [24] to compare 174 chloroplast genomes. As this approach is similar to ours, we compare our tool with their software.

Here we present CNIDARIA, an algorithm that employs a novel RFM strategy for species identification based on *k*-mer counting, designed from the ground up to allow analysis of very large collections of genome, transcriptome and raw NGS data using minimal resources. CNIDARIA improves over previous methods and overcomes their limitations on size and phylogenetic distance by allowing fast analysis of complete NGS data. To this end, it can export a database with pre-processed data so that new samples can be quickly compared against a large database of references, without the need to re-process all the data. In contrast to the method proposed by REFERENCEFREE*,* CNIDARIA is much faster, produces smaller files, is able to produce phylogenetic trees and uses the popular and fast *k-*mer count software JELLYFISH [25], allowing for easy integration in existing NGS quality checking pipelines. We demonstrate the performance and capabilities of CNIDARIA by analysing 169 samples, achieving excellent identification accuracy.

## Implementation

CNIDARIA works with both raw sequencing data and assembled data, both from WGS and RNA-seq sources, in any combination. It uses *k*-mers extracted by JELLYFISH [25], a fast *k*-mer counting tool that produces a database of all *k*-mers present in a query sequence. The advantage of JELLYFISH over comparable software is its ability to create a sparse, compressed database in which the *k*-mers are ordered according to a deterministic hashing algorithm, thus allowing for the parallel and efficient merging/processing of the databases since all *k*-mers are in the same predictable order across different databases. CNIDARIA performs a parallel merge of the sorted sparse databases created by JELLYFISH, creating another sparse database containing, for each *k*-mer, its sequence and a fixed size binary array indicating its presence/absence in each sample. For parallelization, as the number of possible *k-mers* is 4^*k*^, where *k* is the *k*-mer size, each instance of CNIDARIA processes all *k*-mers corresponding to the range $\left[\left(p-1\right)*\frac{4^k}{n},p*\frac{4^k}{n}\right]$ for *p* = 1, …, *n*, with *n* equal to the total number of instances. The partial databases created by each CNIDARIA instance can then simply be concatenated to create a full database containing all *k*-mers.

While merging the JELLYFISH databases into a single database, CNIDARIA extracts the number of *k*-mers shared between each pair of samples and then uses this information to calculate the distance between the samples. For that we used, by default, the *Jaccard* distance as described in CO-PHYLOG [17]:

$$ {D}_{Jaccard}\left({s}_i,{s}_j\right) = 1-\frac{V_{ij}}{V_i+{V}_j-{V}_{ij}} $$

Here, *V*_*ij*_ is the number of *k*-mers shared by both samples *s*_*i*_ and *s*_*j*_, *V*_*i*_ is the number of *k*-mers in sample *s*_*i*_ and *V*_*j*_ is the number of *k*-mers in sample *s*_*j*_. When *s*_*i*_ is equal to *s*_*j*_, the distance is 0. In our implementation, we use the number of valid *k*-mers in a sample, i.e. *k*-mers shared with at least one other sample, to filter out uninformative and possibly erroneous *k*-mers. Please notice that the *k-mer* frequency of each sample is ignored and only their presence/absence used, allowing us to compare divergent sequencing coverage, assembly statuses (from raw data to fully assembled) and sources (DNA or RNA). Besides the Jaccard metric, 70 other distance measures are also implemented in the package.

**Fig. 1:**
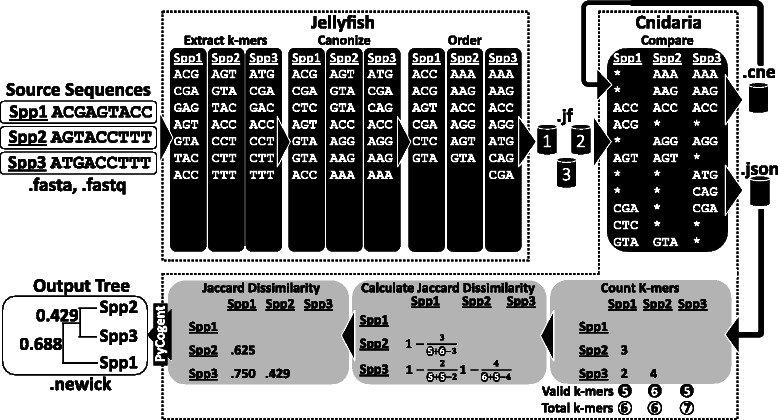
Cnidaria analysis summary. The JELLYFISH software reads each of the source sequence files (in Fasta or Fastq formats), extracts their *k*-mers (*k* = 3 in this example), canonizes them (by generating the reverse complement of each *k*-mer and storing only the *k*-mer which appears first lexicographically), orders them according to a deterministic hashing algorithm (in this example, alphabetically) and then saves each dataset in a separated database file (.jf). CNIDARIA subsequently reads these databases and compares them, side-by-side, by counting the total number of *k*-mers (white circles), the number of valid *k*-mers (*k*-mers shared by at least two samples, black circles) and the number of shared *k*-mers for each pair of samples as a matrix. Those values are exported to a Cnidaria Summary Database (CSD, a .json file) that is then used to construct a matrix of, by default *Jaccard*, distances between the samples (Formula 1). This dissimilarity matrix is then used for Neighbour-Joining clustering and exported as a NEWICK tree. Alternatively, Cnidaria can export a Cnidaria Complete Database (CCD, a .cne file) containing all *k*-mers and a linked list describing their presence/absence in the samples. This second database can be used as an input dataset together with other .cne or .jf files for new analysis

The resulting distance matrix is then processed by PYCOGENT v.1.5.3 [26], which clusters the data using Neighbour-Joining and creates a phylogenetic tree in NEWICK format. For easy visualization of the data, the summary database can also be converted to a standalone HTML page for (dynamic) display of the phylogenetic tree and plotting any statistics of the analysis directly in the tree. A graphical representation of these steps can be found in Fig. 1.

CNIDARIA can be run in two modes: Sample Analysis Mode and Database Creation Mode. Sample Analysis Mode generates a Cnidaria Summary Database (CSD) containing the total number of *k*-mers for each sample, the number of *k*-mers shared by at least two samples (valid *k*-mers), and the pairwise number of shared *k*-mers. Database Creation Mode is an order of magnitude slower than the Sample Analysis Mode but, besides generating the same CSD file, it also exports a Cnidaria Complete Database (CCD). The CCD file contains all *k*-mers present in the datasets analysed, stored in using a two bits per nucleotide encoding (same as JELLYFISH), and their respective presence/absence list. The CCD can be used as an input to CNIDARIA itself in both modes, allowing new samples to be directly compared against a pre-calculated larger dataset, speeding up the analysis significantly since the speed of CNIDARIA is directly correlated to the number and size of the input files. Hence, the software permits a shorter run time for the comparison of a new sample, using Sample Analysis Mode, against a large reference panel stored in a single larger CCD file.

## Results and discussion

### Data set

To validate the performance of CNIDARIA, we gathered a collection of 135 genomic, transcriptomic and raw NGS datasets covering a wide range of organisms. A list of all samples can be found in Additional file 1: Table S1 [27–79]. All datasets were analysed using JELLYFISH with canonized *k*-mers. Canonization is the process of storing the lexicographically smallest *k*-mer between a *k-*mer and its reverse complement. This step is required as both molecules are technically the same: the existence of one implies the existence of the other on the complementary DNA strand. The datasets were then split in 50 pieces and divided over 20 threads on an 80 core Intel(R) Xeon(R) CPU E7- 4850 @ 2.00 GHz machine, speeding up the analysis approximately 40 times compared to single-thread analysis on the same machine. We then created a Cnidaria Complete Database (CCD) containing all 135 samples. *K*-mer counts, *k*-mer statistics and Jaccard distances can be found in Additional file 2: Table S2, Additional file 3: Table S3 and Additional file 4: Table S4, respectively.

### Identification accuracy

**Fig. 2:**
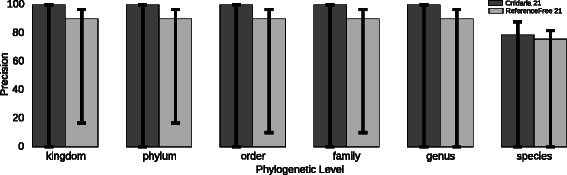
1-nearest-neighbour analysis for species and supra-species levels at each taxonomic level for CNIDARIA and REFERENCEFREE using 21-mers and *Jaccard* distance. Supra-species level analysis contains 30 samples (Additional file 5: Table S5) from 8 genus, 7 families, 7 orders, 4 phylum and 3 kingdoms. Species level analysis contains 33 samples (Additional file 5: Table S5) from 11 species of the Solanum clade. Classification reports the Leave-One-Out Cross-Validation error estimate (LOOCV) for 21-mers. Error bars indicate the minimum and maximum performance found across the 71 distance metrics tested

To verify the accuracy of the clustering of the samples, we used the 1-nearest neighbour algorithm on 30 samples for supra-species level analysis (8 genus, 7 families, 7 orders, 4 phylum and 3 kingdoms, described in Additional file 5: Table S5) and on 33 samples for species level analysis (11 species of the Solanum clade, described in Additional file 6: Table S6). The 1-nearest neighbour classifier reports the percentage of samples for which the sample with the smallest distance belongs to the same rank at each phylogenetic level (species, genus, family, order, phylum and kingdom). We report the percentage of samples correctly classified in Fig. 2 and Additional file 7: Table S7.

### Influence of k-mer size

To investigate the influence of the *k*-mer size on the accuracy of the phylogenetic inference of CNIDARIA, we analysed the panel of 135 samples with *k* = 11, 15, 17, 21 and 31 (predefined hash sizes of 128 million, 256 million, 512 million, 1 billion and 4 billion, respectively). The resulting statistics can also be found in Additional file 1: Table S1.

Due to the low complexity of 11-mers, all possible *k*-mer of this size were found in the datasets and all *k*-mers were valid, i.e. shared by at least two samples (Table 1). This carries little clustering information and generates many zero distances (minimum dissimilarity) as shown in Additional file 8: Figure S1, Additional file 9: Figure S2, Additional file 10: Figure S3, Additional file 11: Figure S4, Additional file 12: Figure S5, Additional file 13: Figure S6 to Additional file 14: Figure S7 and Additional file 4: Table S4. Phylogenetic distances increase with *k*-mer size and 31-mers have most distances equal to 1, i.e. maximum dissimilarity (except for highly related species), which does not allow clustering of distant species. Therefore we chose 21-mers as the default *k*-mer size as it showed the best trade-off between speed and discriminating power (consistent with [23]).

**Table 1:** Summary of search space per *k*-mer size and number of *k*-mers found in datasets

*k*-mer size	# Canonical *k*-mer combinations	% of *k*-mers found per sample		% of *k*-mers found per sample, shared by at least two samples	
		Median	MAD	Median	MAD
11-mer	2.10 × 10^06^	100.00 %	1.58 %	100.00 %	0.00 %
15-mer	5.40 × 10^08^	53.59 %	17.07 %	100.00 %	0.00 %
17-mer	8.60 × 10^09^	8.90 %	4.03 %	98.37 %	0.99 %
21-mer	2.20 × 10^12^	0.05 %	0.03 %	81.45 %	20.55 %
31-mer	2.30 × 10^18^	0.000000061 %	0.000000032 %	67.05 %	24.14 %

The second column contains the total number of possible *k*-mers, calculated as (4^*k*-mer size^/2), where the division by two is due to canonization. The third column is the median and the Median Absolute Deviation (MAD) of the total number of *k-*mers found in the samples (Additional file 3: Table S3) divided by the number of possible *k-*mers, showing the percentage of combinations actually found and, consequently, the saturation of the search space; the fourth column gives the median and MAD of the percentage of valid *k*-mers (*k*-mers shared between at least two samples, Additional file 3: Table S3)

15-mers and 17-mers yielded, at the supra-species level, accuracy above 70 and 90 %, respectively, but below 75 % at the species level. Both 21- and 31-mers allowed us to correctly classify 100 % of the samples at the supra-species level and 78 % at the species level (Additional file 7: Table S7). The lower accuracy for species level classification in the tomato clade can be attributed to introgressions and sympatric speciation in tomato and is in agreement with the clustering obtained by [27], which used whole genome SNP analysis to construct trees. Compared to using 21-mers, the use of 31-mers resulted in an increased run time and disk usage without yielding a discernibly higher discriminative power. This suggests 21 is a good *k*-mer size for general purpose clustering. However, 31-mers are frequently used for NGS data quality checking (reviewed in [80]) and the same JELLYFISH database created for quality checking can be used for species identification.

### Influence of distance measure

In order to identify the best distance measure to apply, 71 binary distances measures were implemented in CNIDARIA according to [81] and the results can be found in Additional file 7: Table S7. Some measures gave a better sensitivity than the *Jaccard* distance at shorter *k*-mer lengths, but in these cases the accuracy was below 100 %. At *k* = 21, *Jaccard* distance presented an overall high accuracy, although other methods achieved similar results. We decided to use *Jaccard* as the default measure due to its simplicity and equally high accuracy as other methods.

### Joint analysis of DNA and RNA-seq data

**Fig. 3:**
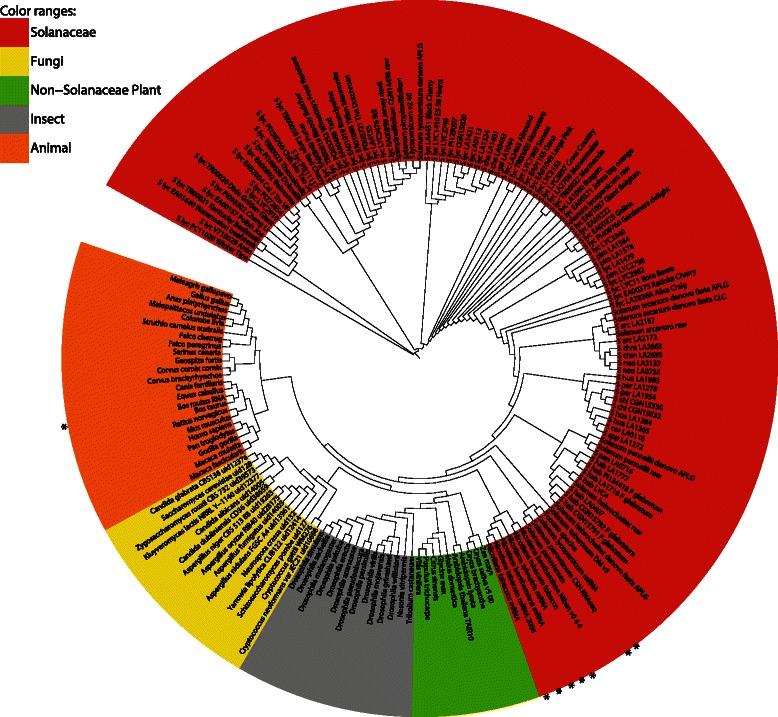
Results for the 21-mer dataset of 169 individuals using the *Jaccard* distance and Neighbour-Joining. The phylogenetic tree shows the clustering of the samples without displaying branch lengths (plotted using iTOL, [85]). RNAseq samples are highlighted with a * in the outer rim of the tree

Next, we expanded the 135 sample dataset (built using Database Creation Mode) with 34 extra samples, 26 genomic and 8 RNA-seq (Additional file 1: Table S1), using 21-mers and the faster Sample Analysis Mode. RNA-seq samples were added to verify whether transcriptome data would cluster with their genomic NGS counterparts, despite their small coverage of the genome length. Results are shown in Fig. 3 and Additional file 15: Figure S8. The clustering of the original 135 samples is not changed and new samples cluster correctly according to their phylogeny. The consistent clustering observed for the RNA-seq dataset illustrates the ability of CNIDARIA to use such data for accurate species identification.

### Speedup by subsampling

To test the influence of data set size (and possibility of speedup) we sample 2 % of the 21-mer dataset, by analysing just 1 of the 50 pieces the data was originally split into. Additional file 16: Figure S9 shows the phylogenetic placement of species in the trees constructed using this dataset and Additional file 7: Table S7 shows the classification accuracy. The tree is indistinguishable from the one generated on the full dataset, illustrating the ability of CNIDARIA to correctly classify samples even at very low sequencing coverage. This suggests that CNIDARIA should be able to correctly cluster and identify samples using small and affordable NGS sequencing technology such as Illumina MiSeq nano runs (500 Mbp in 2 × 250 bp reads, [82]).

### Comparison with REFERENCEFREE

To demonstrate the advantages of CNIDARIA, we compare it to a state-of-the-art tool called REFERENCEFREE [23]. Its latest version (1.1.3) was downloaded and run in conjunction with ABYSS [83] version 1.3.3. We run this older version rather than the latest version (1.9.0) since that was the version REFERENCEFREE was designed to work with. REFERENCEFREE was run single threaded on an Intel(R) Xeon(R) CPU E7- 4850 @ 2.00 GHz with a *k*-mer size of 21, a minimum frequency of 0 (i.e. using all *k*-mers appearing 1 or more times), no complexity filter and no sampling of *k*-mers. The list of shared *k*-mers generated was then parsed using the CNIDARIA scripts in order to generate a comparable phylogenetic tree, since REFERENCEFREE does not provide a method for phylogenetic analysis.

Using a subset of our data (41 assembled genomes, Additional file 1: Table S1) containing 40 Gbp and 20 billion *k*-mers, REFERENCEFREE (Additional file 1: Table S1) and JELLYFISH have a comparable speed for *k*-mer counting, taking 4 h to count 445 million *k-*mers (2 % of the total; Additional file 17: Table S8). REFERENCEFREE then took 60 % more time than CNIDARIA in single threaded Sample Analysis Mode for merging and summarizing the results (70 h vs. 44 h, respectively). Note that the databases created by CNIDARIA can be re-used in subsequent comparisons, whereas REFERENCEFREE requires all the *k*-mer count files to be merged again when re-run. Moreover, CNIDARIA has the important advantage of being highly parallelizable while REFERENCEFREE can only be run single threaded.

Regarding accuracy, Fig. 2 shows that REFERENCEFREE, using *Jaccard* distance and 21-mers, was slightly less accurate than CNIDARIA, although it can achieve comparable results with different distance measures (Additional file 7: Table S7). Besides speed, CNIDARIA (and JELLYFISH) use significantly less disk space due to their binary formats. The files generated are smaller than the equivalent files created by REFERENCEFREE, with median sizes of 9.2 Gb vs. 42.2 Gb (and median absolute deviations of 2.5 Gb and 11.0 Gb, respectively) for the *k*-mer count file and 227 Gb vs. 2.1 Tb for the merged *k*-mer count file, despite the merged *k*-mer count file created by REFERENCEFREE containing only 2 % of the total number of *k*-mers, all of which are present in CNIDARIA.

## Conclusions

We have introduced CNIDARIA, a tool to quickly and reliably analyse WGS and RNA-seq samples from both assembled and unassembled NGS data, offering significant advantages in terms of time and space requirements compared to a state-of-the-art tool. By clustering in total 169 eukaryotic samples from 78 species (42 genus, 32 families, 27 orders, 5 phyla, 6 divisions and 3 kingdoms from the Eukaryota superkingdom) we have demonstrated that CNIDARIA can handle a large number of samples from very distant phylogenetic origins, producing a reliable tree with up to 100 % classification accuracy at the supra species level and 78 % accuracy at the species level, the later value being low mostly due to interspecific crossings. As CNIDARIA is also able to analyse RNA-seq data, researchers can acquire, besides the species information, physiological state information such as pathogenicity and stress response of the sample for downstream analysis.

A database created in Database Creation Mode allows querying directly for *k*-mers shared by a specified set of samples, enabling comparisons useful in several applications. Examples include identifying and quantifying polymorphisms between closely related samples, quantifying sequence diversity in the setup phase of large sequencing projects for sample selection, and ecological diversity analysis. In addition, *k*-mers shared exclusively by a set of samples can be used for diagnostic primer design, supporting the detection of target genes. Furthermore, mismatching *k*-mers between a sample and a close relative can be used to identify the source of contamination or introgressions, as performed by [84].

## Availability and requirements

Project name: Cnidaria

Project home page:  http://www.ab.wur.nl/cnidaria;  https://github.com/sauloal/cnidaria/wiki

Operating system(s): 64-bit Linux

Programming language: C++ ×11 and Python 2.7

Other requirements: None to run; GCC 4.8 or higher for compiling

License: MIT

Any restrictions to use by nonacademics: No

## Additional files

Additional file 1: Table S1. Sample description. Intermediate headers show database size and analysis time running with 1 thread (1×) or 20 threads (20×) for each CNIDARIA database group. Each line contains a list of the names of the samples used, sequence ID, source type, source name, reference, size of JELLYFISH database, size of input data, GC content, percentage of Ns, number of sequences in the input data and list of samples used in the REFERENCEFREE comparison. For each *k*-mer size (11, 15, 17, 21 and 31 bp): number of distinct *k*-mers, total number of *k*-mers, number of *k*-mers occurring only once, number of shared *k*-mers and percentage of *k*-mers shared. Input data is in the form of assembled genome (genomic - fasta files), raw genomic data (raw - fastq or BAM), filtered genomic data (raw filtered - BAM) or RNA-seq. The 34 samples of the extended dataset were used exclusively against the 21-mer dataset. Analysis time and database sizes are calculated for each dataset and do not correspond to the sum of the partial times and sizes. (XLS 87 kb)
Additional file 2: Table S2. Matrix containing the pairwise number of shared *k*-mers. The diagonal contains the number of valid *k*-mers (*k*-mers shared with at least 1 other sample) of a given sample. 11, 15, 17, 21 and 31-mers are shown as well as the 21-mer dataset downsampled to 2 % of its original size and 21-mer dataset with 34 extra samples. (XLS 1217 kb)
Additional file 3: Table S3. Statistics of *k*-mer counting. Total number of *k*-mers in each sample, total number of valid *k*-mers in each sample (*k*-mers shared by at least two samples) and the percentage of valid *k*-mers in each sample. 11, 15, 17, 21 and 31-mers are shown as well as the 21-mer dataset downsampled to 2 % of its original size and 21-mer dataset with 34 extra samples. (XLS 104 kb)
Additional file 4: Table S4. Matrix containing pairwise *Jaccard* distance between samples. The diagonal is blanked but contains zeroes, meaning identity; 11, 15, 17, 21 and 31-mers are shown, as well as the 21-mer dataset downsampled to 2 % of its original size and 21-mer dataset with 34 extra samples. (XLS 2617 kb)
Additional file 5: Table S5. List of samples used for the 1-nearest-neighbour analysis for supra-species classification and their respective taxonomic ranks. (XLS 29 kb)
Additional file 6: Table S6. List of samples used for the 1-nearest-neighbour analysis for species classification and their respective taxonomic ranks. (XLS 26 kb)
Additional file 7: Table S7. 1-nearest-neighbour accuracy results for all *k-*mer sizes, distance measures and programs. CNIDARIA is tested against all *k*-mer sizes. REFERENCEFREE is tested using 21-mers. Cnidaria 21 2 % is the dataset containing only 2 % of the data. (XLS 91 kb)
Additional file 8: Figure S1. Histogram of *Jaccard* distances for each *k*-mer size of the 135 samples. A distance of 0 means identity while a distance of 1 means no similarity. Using 11-mers most samples are identical to each other. For 31-mers, most samples share no similarity with any other sample except for phylogenetically closely related samples. 17 and 21-mers show higher similarity between groups. (PDF 97 kb)
Additional file 9: Figure S2. Heatmaps of *Jaccard* distance and phylogenetic trees of 135 samples using 11-mers. Here, 0 (red) means identity between samples while 1 (blue) means no identity. Generally, closely related species show high similarity with closely related species and no similarity with outgroups. This leads to strong clustering inside groups but loose coupling between groups. Trees in the left shows phylogenetic distances while trees in the right ignores the distances, showing the clustering more clearly; trees plotted using iTOL [85]. (PDF 1668 kb)
Additional file 10: Figure S3. Heatmaps of Jaccard distance and phylogenetic trees from 135 samples using 15-mers. Here, 0 (red) means identity between samples while 1 (blue) means no identity. Generally, closely related species show high similarity with closely related species and no similarity with outgroups. This leads to strong clustering inside groups but loose coupling between groups. Trees on the left shows phylogenetic distances while trees on the right ignores the distances, showing the clustering more clearly. Trees were plotted using iTOL [85]. (PDF 1499 kb)
Additional file 11: Figure S4. Heatmaps of Jaccard distance and phylogenetic trees from 135 samples using 17-mers. Here, 0 (red) means identity between samples while 1 (blue) means no identity. Generally, closely related species show high similarity with closely related species and no similarity with outgroups. This leads to strong clustering inside groups but loose coupling between groups. Trees on the left shows phylogenetic distances while trees on the right ignores the distances, showing the clustering more clearly. Trees were plotted using iTOL [85]. (PDF 1363 kb)
Additional file 12: Figure S5. Heatmaps of Jaccard distance and phylogenetic trees from 135 samples using 21-mers. Here, 0 (red) means identity between samples while 1 (blue) means no identity. Generally, closely related species show high similarity with closely related species and no similarity with outgroups. This leads to strong clustering inside groups but loose coupling between groups. Trees in the left shows phylogenetic distances while trees in the right ignores the distances, showing the clustering more clearly; trees plotted using iTOL [85]. (PDF 1303 kb)
Additional file 13: Figure S6. Heatmaps of Jaccard distance and phylogenetic trees from 135 samples using 31-mers. Here, 0 (red) means identity between samples while 1 (blue) means no identity. Generally, closely related species show high similarity with closely related species and no similarity with outgroups. This leads to strong clustering inside groups but loose coupling between groups. Trees on the left shows phylogenetic distances while trees on the right ignores the distances, showing the clustering more clearly. Trees were plotted using iTOL [85] (PDF 1292 kb)
Additional file 14: Figure S7. Phylogenetic tress with and without branch lengths of 98 *Solanum* taxa from 13 species. The *Lycopersicon* group (comprised of *Solanum lycopersicum*, *S pimpinellifolium*, *S. cheesmaniae* and *S. galapagense*) clusters as a monophyletic group. Sometimes the non-*S. lycopersicum* species cluster inside the *S. lycopersicum* clade. We speculate these are *S. lycopersicum* varieties containing introgression clustering with the donor species, consistently with the findings of [27]. The *Arcanum* group (comprised of *S. arcanum*, *S. chmielewskii* and *S. neorikii*) also clusters monophyletically, closer to the *Eriopersicon* group, its sister group. The North *Eriopersicon* group (comprised of *S. huaylasense*, *S. chilense*, *S. peruvianum* and *S. corneliomulleri*) groups with the South *Eriopersicon* group (comprised of *S. habrochaites*, its only member) and its sister group, *Neolycopersicon* (comprised of *S. pennelli*, its only member). *S. tuberosum* and *Nicotiana* were added as outgroups. Sample names ending in RAW are raw genomic data; names ending in APLG and CLC are assembled genomes. Trees were plotted using iTOL [85] (PDF 1862 kb)
Additional file 15: Figure S8. Results for the 21-mer dataset of 169 individuals using *Jaccard* distance and Neighbour-Joining. (A) phylogenetic tree with distance; (B) phylogenetic tree without distance (tree branch length); (C) heatmap of phylogenetic distances showing low inter-group similarity and high intra-group similarity; (D) histogram of *Jaccard* distances showing the same feature of low inter-group similarity and high intra-group similarity. Sample names ending in RAW are raw genomic data; names ending in APLG and CLC are assembled genomes; names ending in RNA, RNAseq and mRNA are RNA-seq datasets. Trees were plotted using iTOL [85] (PDF 2029 kb)
Additional file 16: Figure S9. Results for 2 % of the 21-mer dataset. (A) phylogenetic tree with distance; (B) phylogenetic tree without distance; (C) heatmap of phylogenetic distances showing low inter-group similarity and high intra-group similarity; (D) histogram of *Jaccard* distances showing the same feature of low inter-group similarity and high intra-group similarity. Trees were plotted using iTOL [85] (PDF 1709 kb)
Additional file 17: Table S8. REFERENCEFREE datasets and statistics. Datasets used in the REFERENCEFREE analysis with the respective number of sequences, number of *k-*mers, number of valid *k-*mers (present in at least two samples) and percentage of *k*-mers considered valid for each dataset. On average, 0.016 ± 0.023 % of the data is used. (XLS 34 kb)

## Abbreviations

CCD: Cnidaria Complete Database
CSD: Cnidaria Summary Database
CSV: Comma-Separated Values
DFT: Discrete Fourier Transform
EST: Expressed Sequence Tag
*K*-mer: Substring of size *k*
MAD: Median Absolute Deviation
NGS: Next Generation Sequencing
PNG: Portable Network Graphics
rDNA: Ribosomal DNA
RFM: Reference-Free Methods
RNA-seq: RNA sequencing
WGS: Whole Genome Sequencing
.fastq: Raw assembly file
.fasta: Assembled sequence
.newick: Phylogenetic tree file format
HTML: HyperText Markup Language
Read: Contiguous sequence outputted by sequencing machine
