# Supplementary figures and images for: Cnidaria: fast, reference-free clustering of raw and assembled genome and transcriptome NGS data
Source: BMC Bioinformatics. 2015 Nov 2;16:352. doi: 10.1186/s12859-015-0806-7 (PMC4630969; doi:10.1186/s12859-015-0806-7)

**11-mer**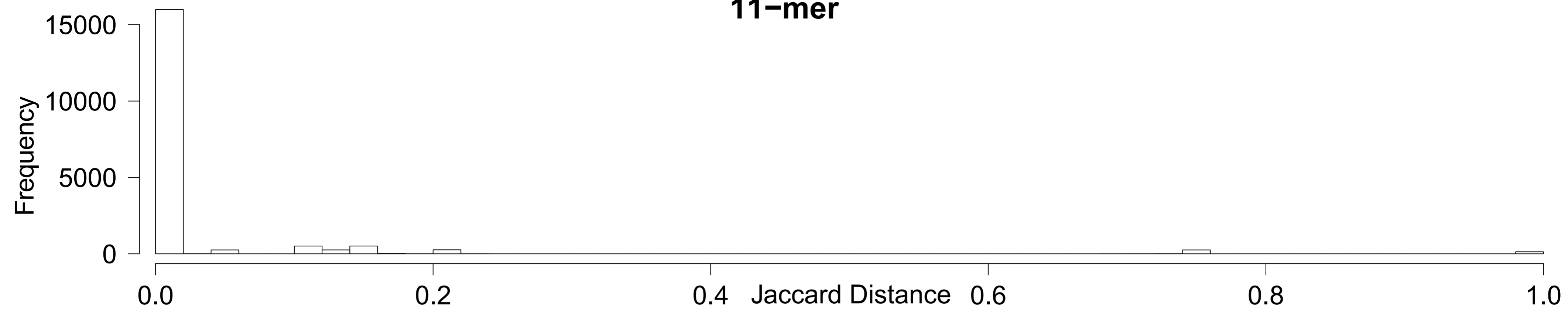**15-mer**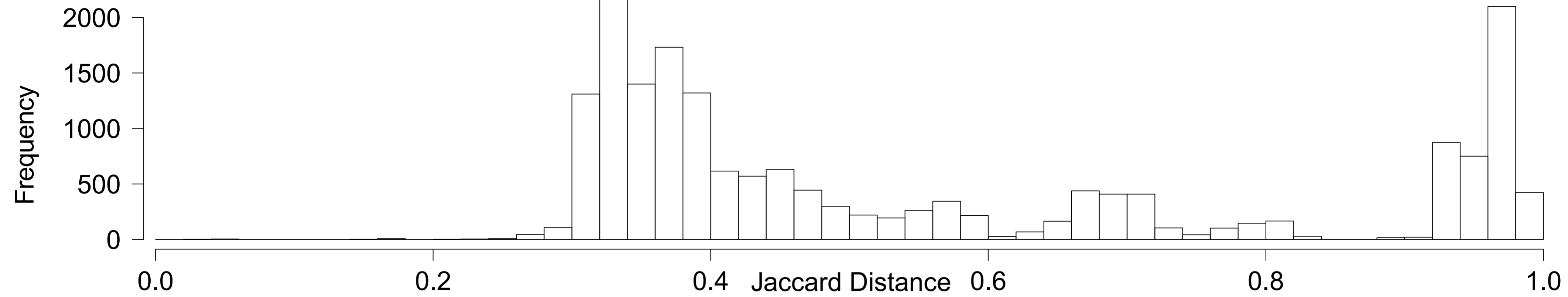**17-mer**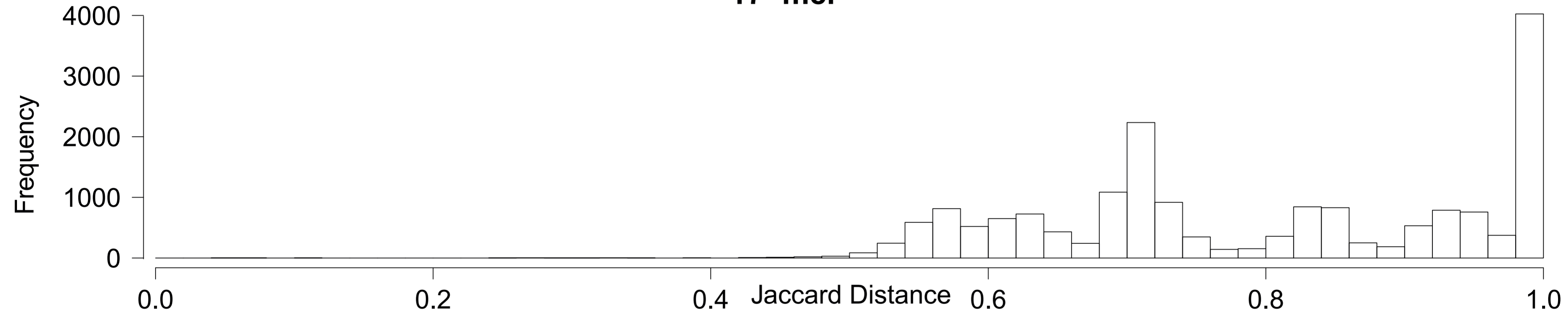**21-mer**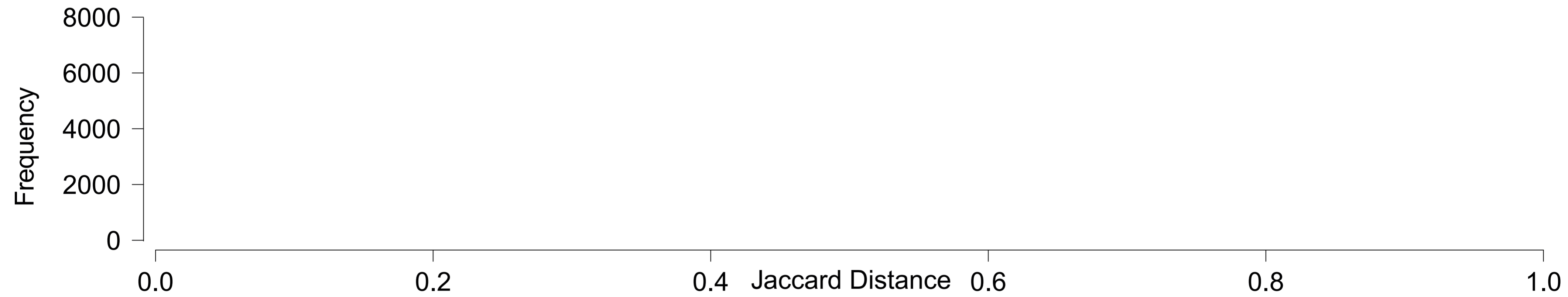**31-mer**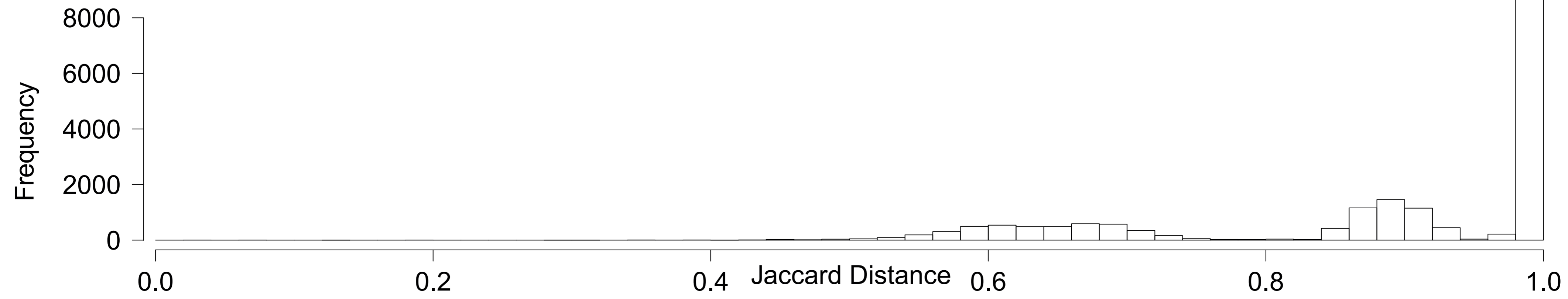

Supplement: Additional file 8: Figure S1. — Histogram of Jaccard distances for each k-mer size of the 135 samples. A distance of 0 means identity while a distance of 1 means no similarity. Using 11-mers most samples are identical to each other. For 31-mers, most samples share no similarity with any other sample except for phylogenetically closely related samples. 17 and 21-mers show higher similarity between groups. (PDF 97 kb) [file 12859_2015_806_MOESM8_ESM.pdf]

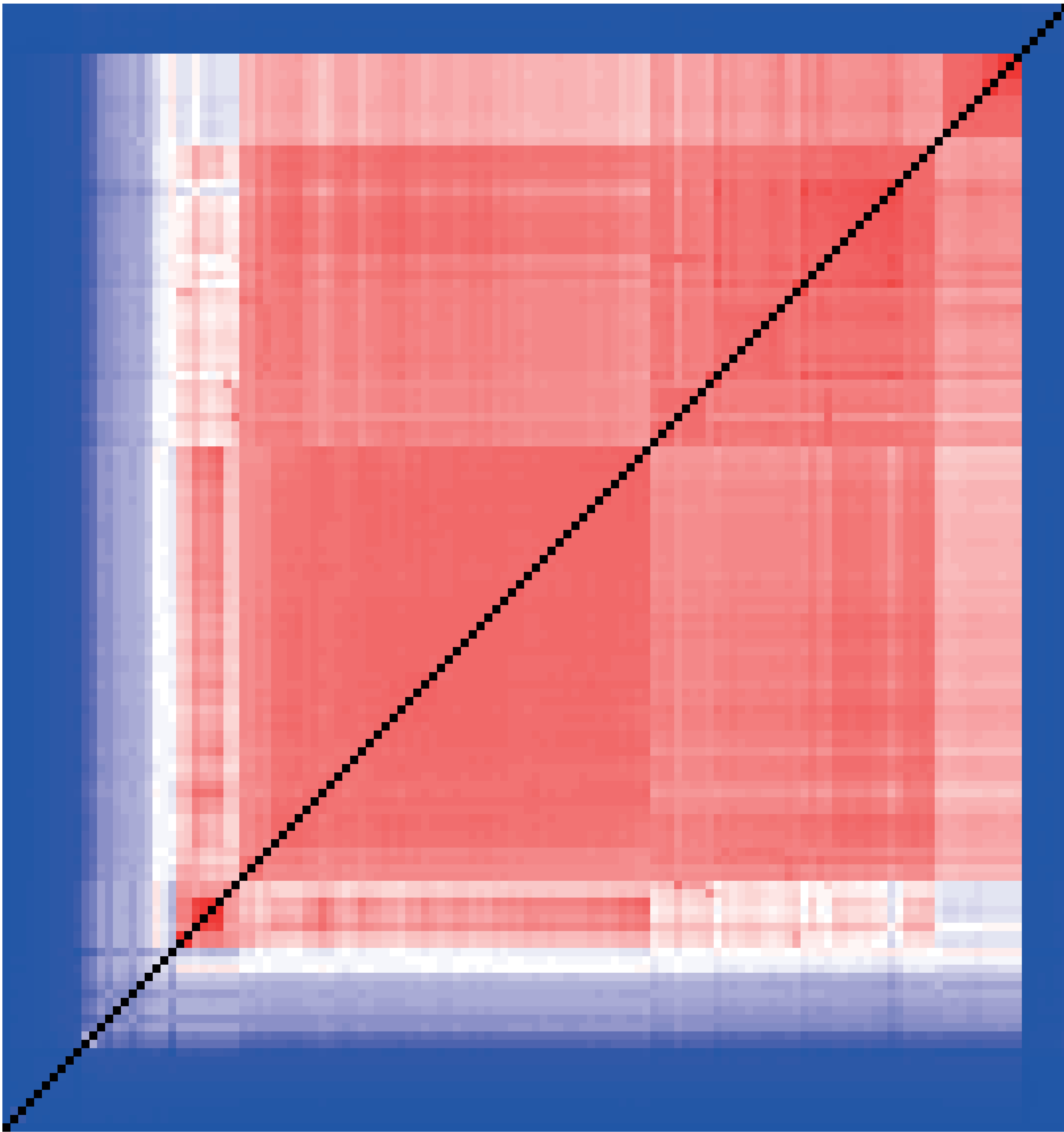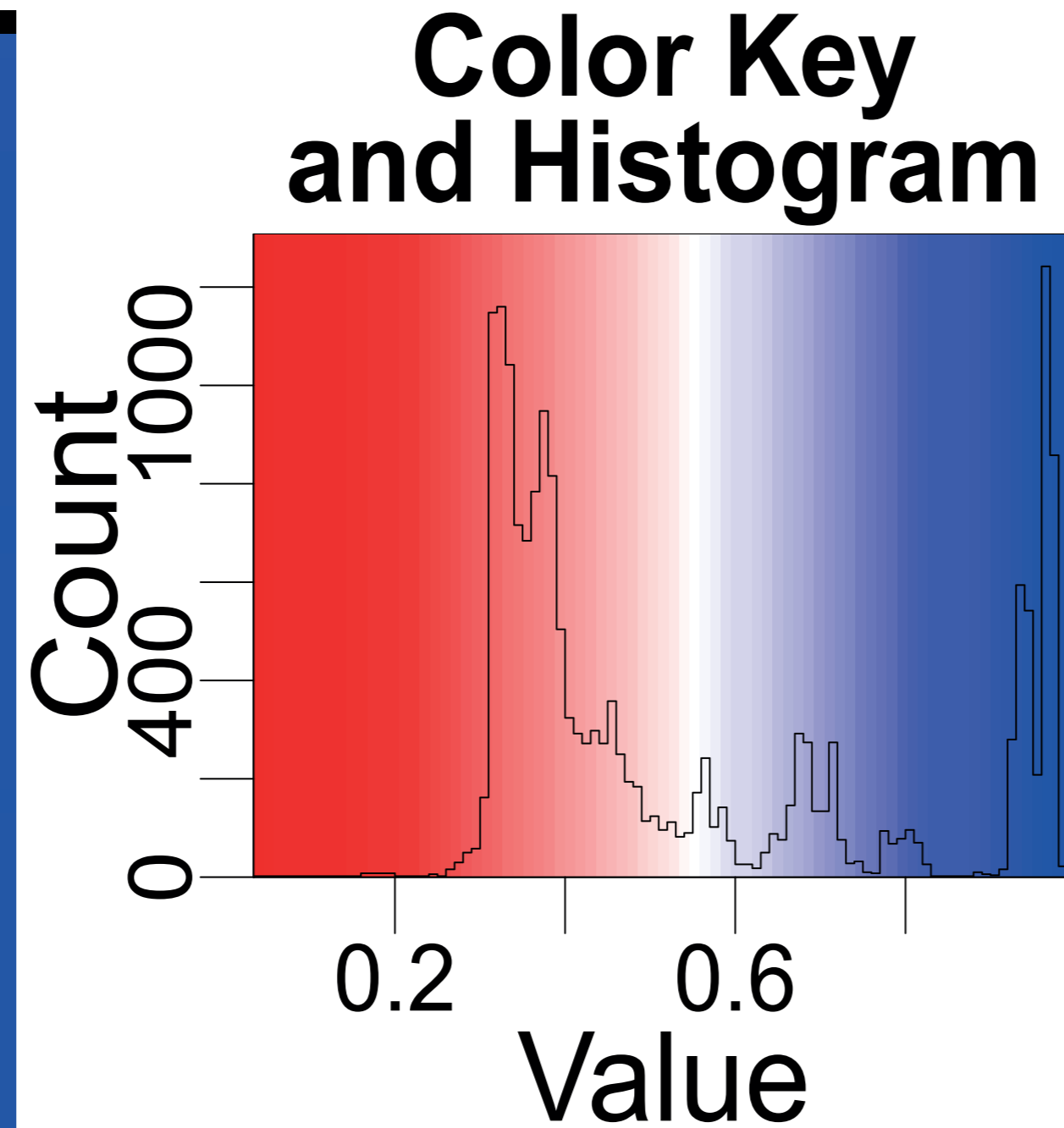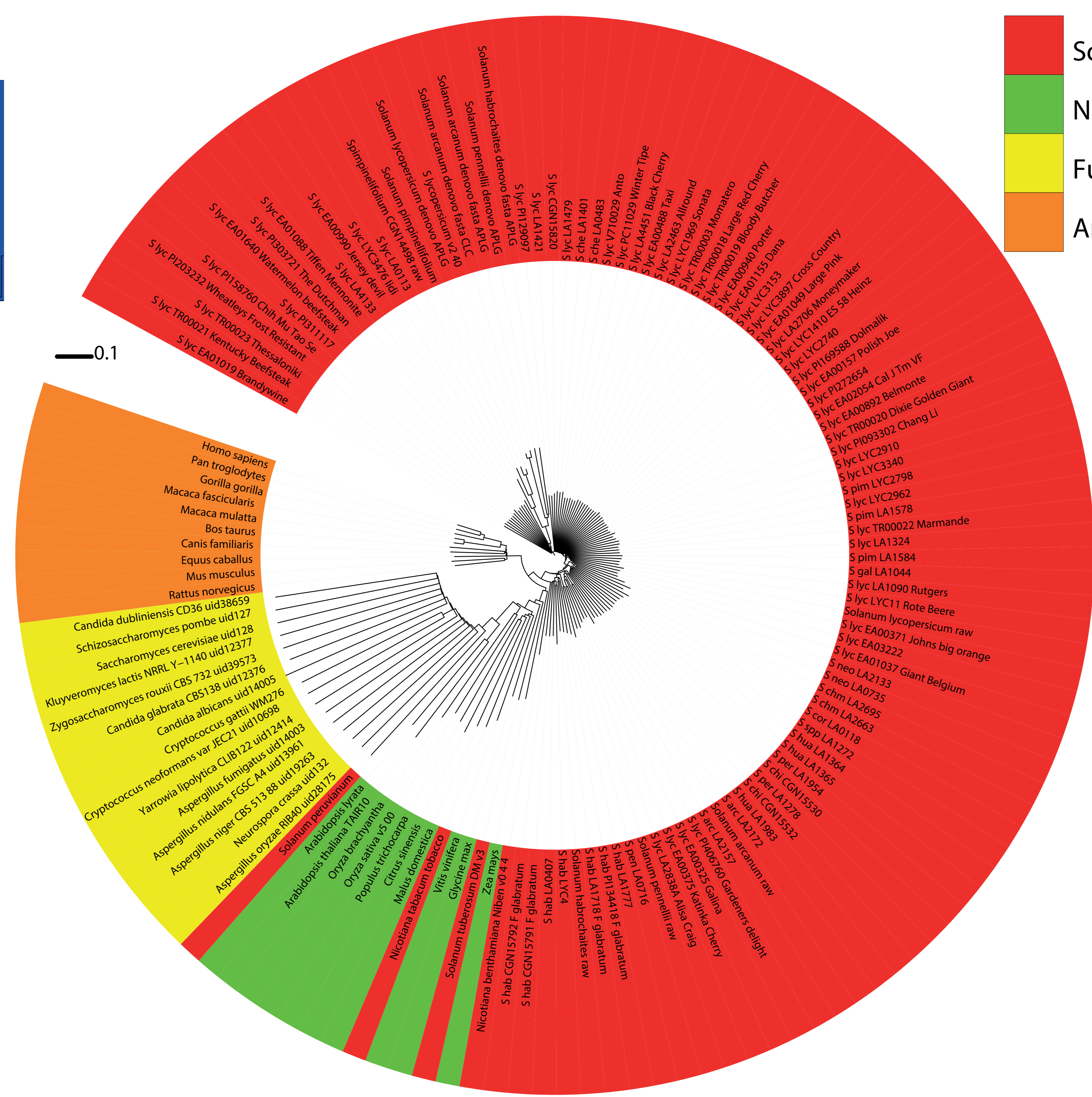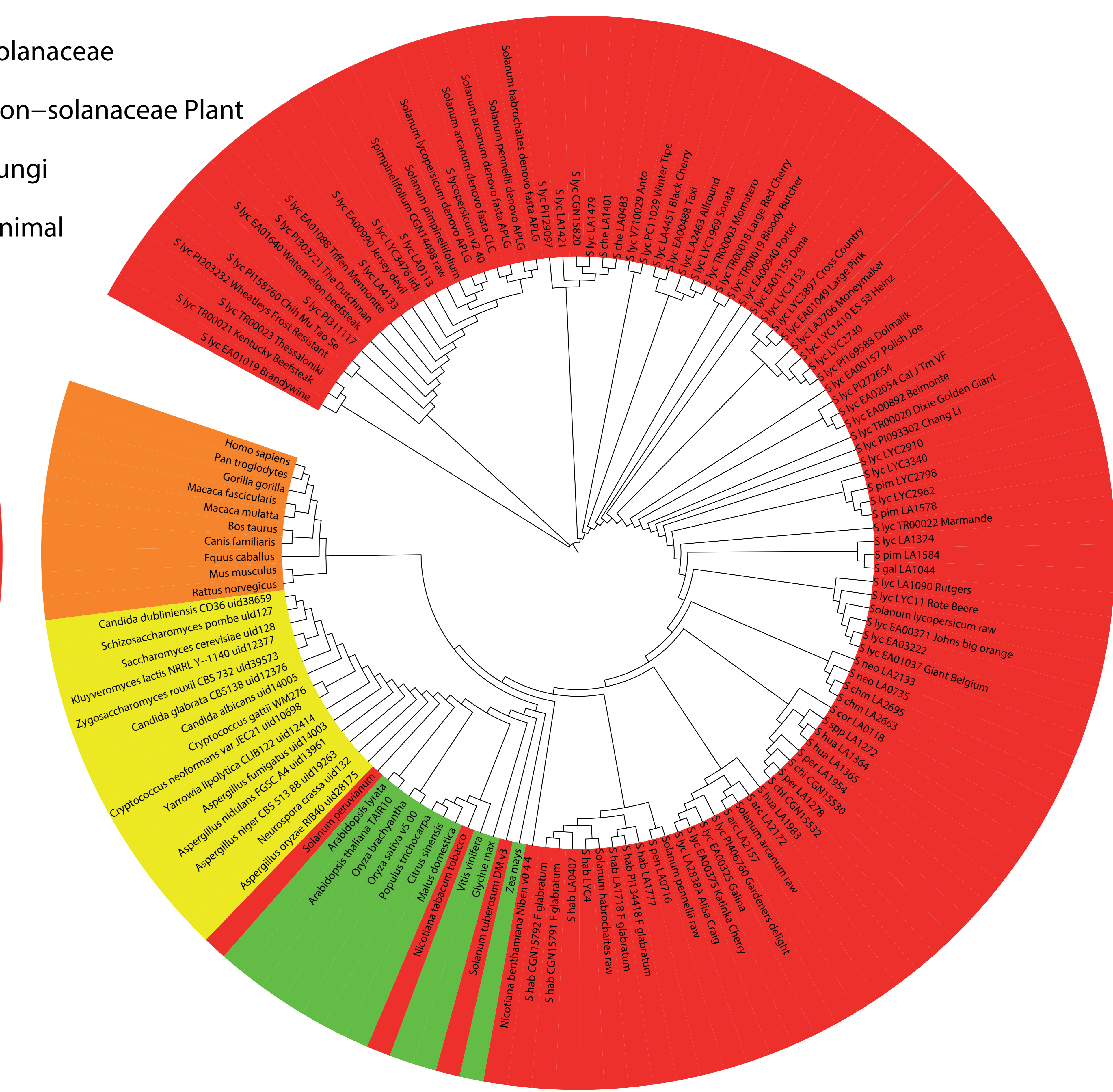

Supplement: Additional file 10: Figure S3. — Heatmaps of Jaccard distance and phylogenetic trees from 135 samples using 15-mers. Here, 0 (red) means identity between samples while 1 (blue) means no identity. Generally, closely related species show high similarity with closely related species and no similarity with outgroups. This leads to strong clustering inside groups but loose coupling between groups. Trees on the left shows phylogenetic distances while trees on the right ignores the distances, showing the clustering more clearly. Trees were plotted using iTOL [85]. (PDF 1499 kb) [file 12859_2015_806_MOESM10_ESM.pdf]

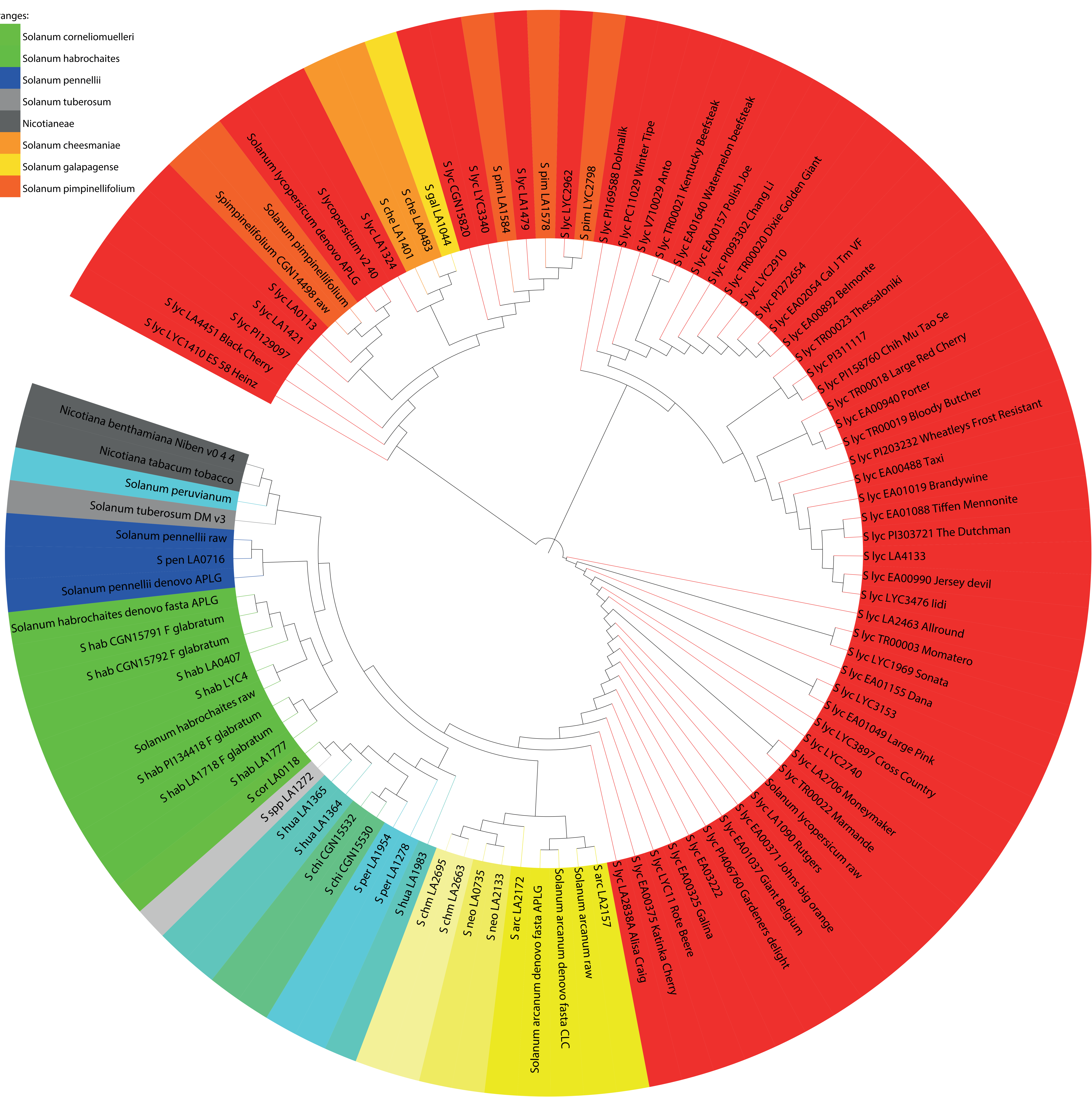

Supplement: Additional file 14: Figure S7. — Phylogenetic tress with and without branch lengths of 98 Solanum taxa from 13 species. The Lycopersicon group (comprised of Solanum lycopersicum, S pimpinellifolium, S. cheesmaniae and S. galapagense) clusters as a monophyletic group. Sometimes the non-S. lycopersicum species cluster inside the S. lycopersicum clade. We speculate these are S. lycopersicum varieties containing introgression clustering with the donor species, consistently with the findings of [27]. The Arcanum group (comprised of S. arcanum, S. chmielewskii and S. neorikii) also clusters monophyletically, closer to the Eriopersicon group, its sister group. The North Eriopersicon group (comprised of S. huaylasense, S. chilense, S. peruvianum and S. corneliomulleri) groups with the South Eriopersicon group (comprised of S. habrochaites, its only member) and its sister group, Neolycopersicon (comprised of S. pennelli, its only member). S. tuberosum and Nicotiana were added as outgroups. Sample names ending in RAW are raw genomic data; names ending in APLG and CLC are assembled genomes. Trees were plotted using iTOL [85] (PDF 1862 kb) [file 12859_2015_806_MOESM14_ESM.pdf]
