# Supplementary material for: Cnidaria: fast, reference-free clustering of raw and assembled genome and transcriptome NGS data
Source: BMC Bioinformatics. 2015 Nov 2;16:352. doi: 10.1186/s12859-015-0806-7 (PMC4630969; doi:10.1186/s12859-015-0806-7)
Supplement: Additional file 9: Figure S2. — Heatmaps of Jaccard distance and phylogenetic trees of 135 samples using 11-mers. Here, 0 (red) means identity between samples while 1 (blue) means no identity. Generally, closely related species show high similarity with closely related species and no similarity with outgroups. This leads to strong clustering inside groups but loose coupling between groups. Trees in the left shows phylogenetic distances while trees in the right ignores the distances, showing the clustering more clearly; trees plotted using iTOL [85]. (PDF 1668 kb) [file 12859_2015_806_MOESM9_ESM.pdf]

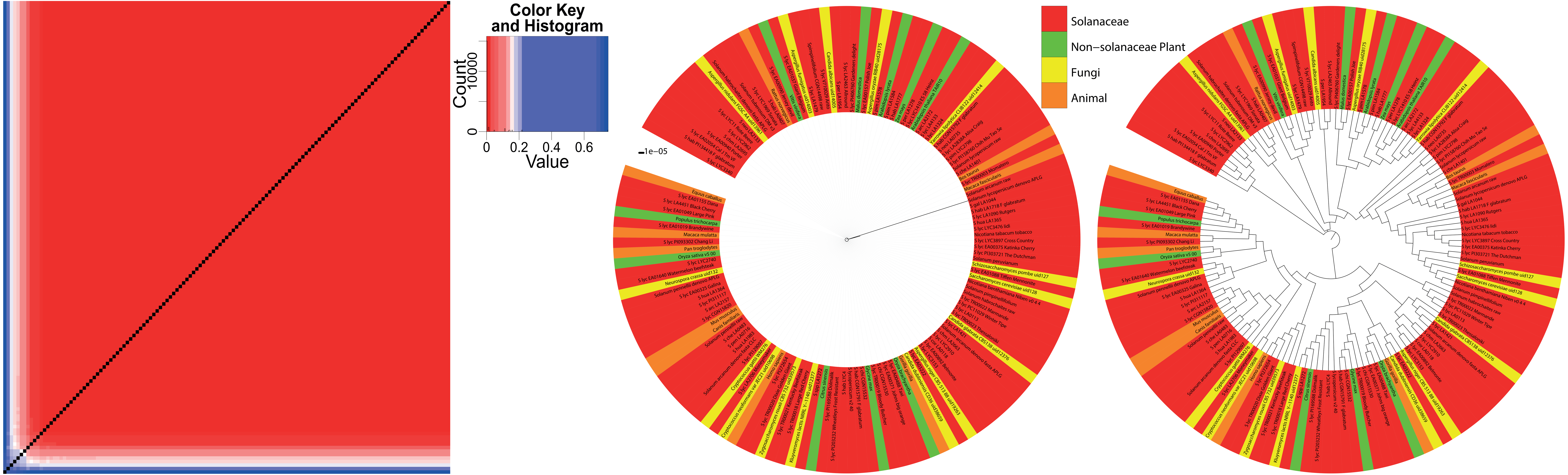
Color Key
and Histogram
Count
0
10000
0
0.2
0.4
0.6
Value
1e−05
Solanaceae
Non−solanaceae Plant
Fungi
Animal
