# Supplementary material for: Cnidaria: fast, reference-free clustering of raw and assembled genome and transcriptome NGS data
Source: BMC Bioinformatics. 2015 Nov 2;16:352. doi: 10.1186/s12859-015-0806-7 (PMC4630969; doi:10.1186/s12859-015-0806-7)
Supplement: Additional file 11: Figure S4. — Heatmaps of Jaccard distance and phylogenetic trees from 135 samples using 17-mers. Here, 0 (red) means identity between samples while 1 (blue) means no identity. Generally, closely related species show high similarity with closely related species and no similarity with outgroups. This leads to strong clustering inside groups but loose coupling between groups. Trees on the left shows phylogenetic distances while trees on the right ignores the distances, showing the clustering more clearly. Trees were plotted using iTOL [85]. (PDF 1363 kb) [file 12859_2015_806_MOESM11_ESM.pdf]

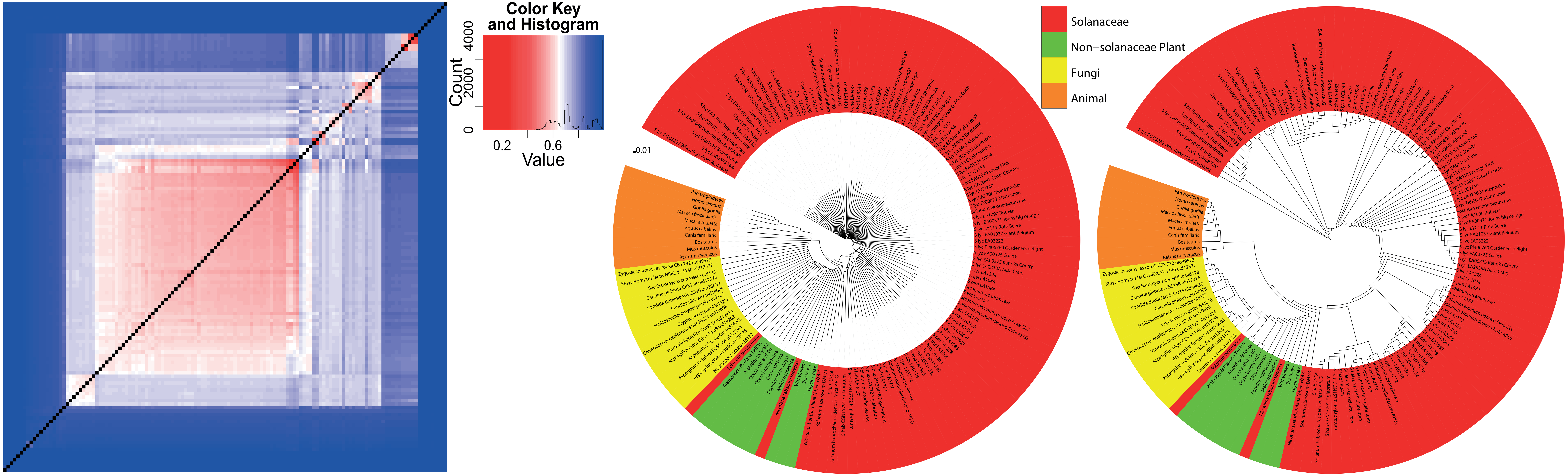

Color Key
and Histogram
Count
0
2000
4000
0.2
0.6
Value
0.01
Solanaceae
Non–solanaceae Plant
Fungi
Animal
