# Supplementary material for: Cnidaria: fast, reference-free clustering of raw and assembled genome and transcriptome NGS data
Source: BMC Bioinformatics. 2015 Nov 2;16:352. doi: 10.1186/s12859-015-0806-7 (PMC4630969; doi:10.1186/s12859-015-0806-7)
Supplement: Additional file 15: Figure S8. — Results for the 21-mer dataset of 169 individuals using Jaccard distance and Neighbour-Joining. (A) phylogenetic tree with distance; (B) phylogenetic tree without distance (tree branch length); (C) heatmap of phylogenetic distances showing low inter-group similarity and high intra-group similarity; (D) histogram of Jaccard distances showing the same feature of low inter-group similarity and high intra-group similarity. Sample names ending in RAW are raw genomic data; names ending in APLG and CLC are assembled genomes; names ending in RNA, RNAseq and mRNA are RNA-seq datasets. Trees were plotted using iTOL [85] (PDF 2029 kb) [file 12859_2015_806_MOESM15_ESM.pdf]

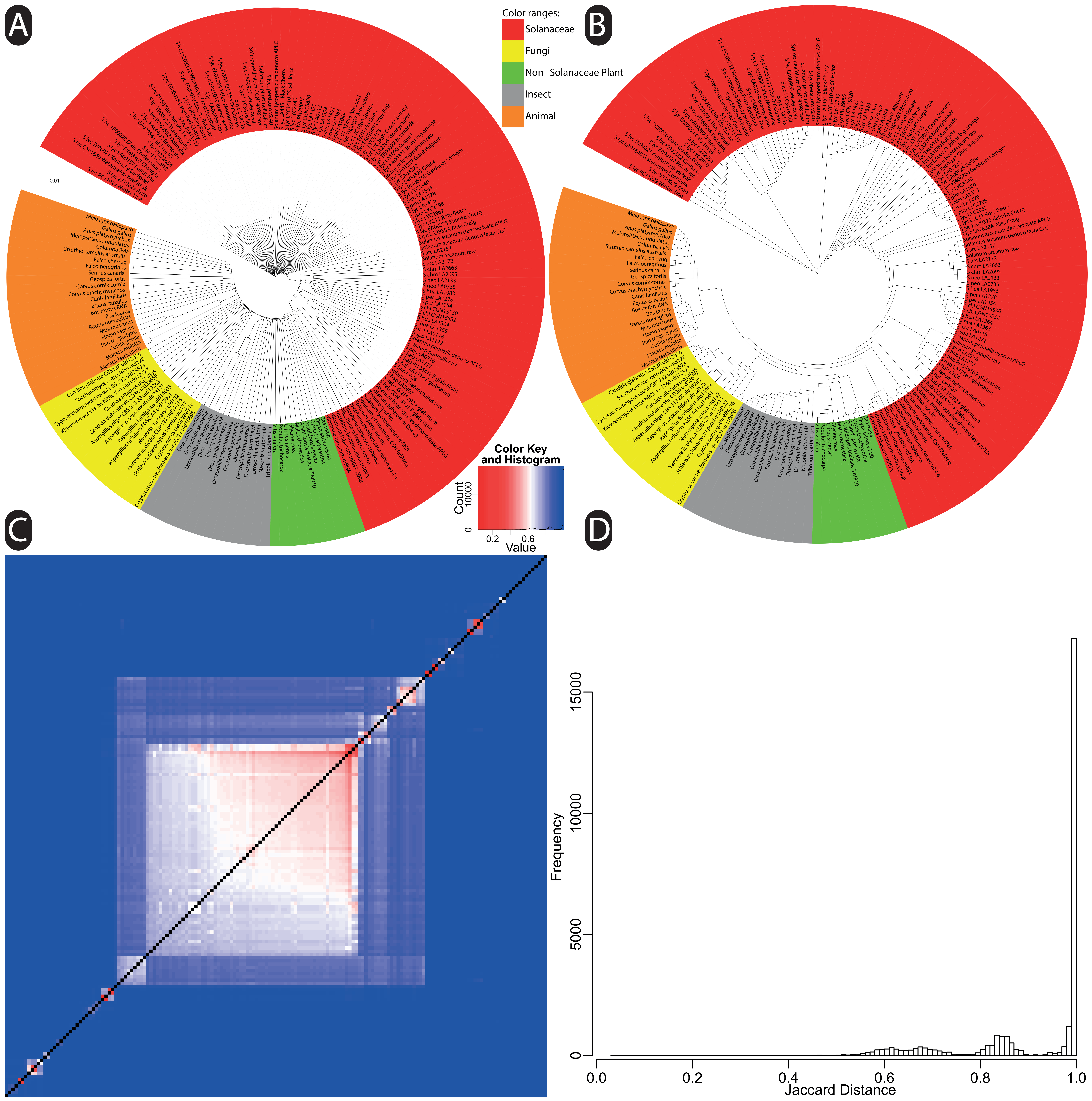
A
B
C
D
Color ranges:
Solanaceae
Fungi
Non–Solanaceae Plant
Insect
Animal
0.01
Color Key and Histogram
Count
0
10000
0.2
0.6
Value
Frequency
0
5000
10000
15000
0.0
0.2
0.4
0.6
0.8
1.0
Jaccard Distance
