# Supplementary material for: Cnidaria: fast, reference-free clustering of raw and assembled genome and transcriptome NGS data
Source: BMC Bioinformatics. 2015 Nov 2;16:352. doi: 10.1186/s12859-015-0806-7 (PMC4630969; doi:10.1186/s12859-015-0806-7)
Supplement: Additional file 16: Figure S9. — Results for 2 % of the 21-mer dataset. (A) phylogenetic tree with distance; (B) phylogenetic tree without distance; (C) heatmap of phylogenetic distances showing low inter-group similarity and high intra-group similarity; (D) histogram of Jaccard distances showing the same feature of low inter-group similarity and high intra-group similarity. Trees were plotted using iTOL [85] (PDF 1709 kb) [file 12859_2015_806_MOESM16_ESM.pdf]

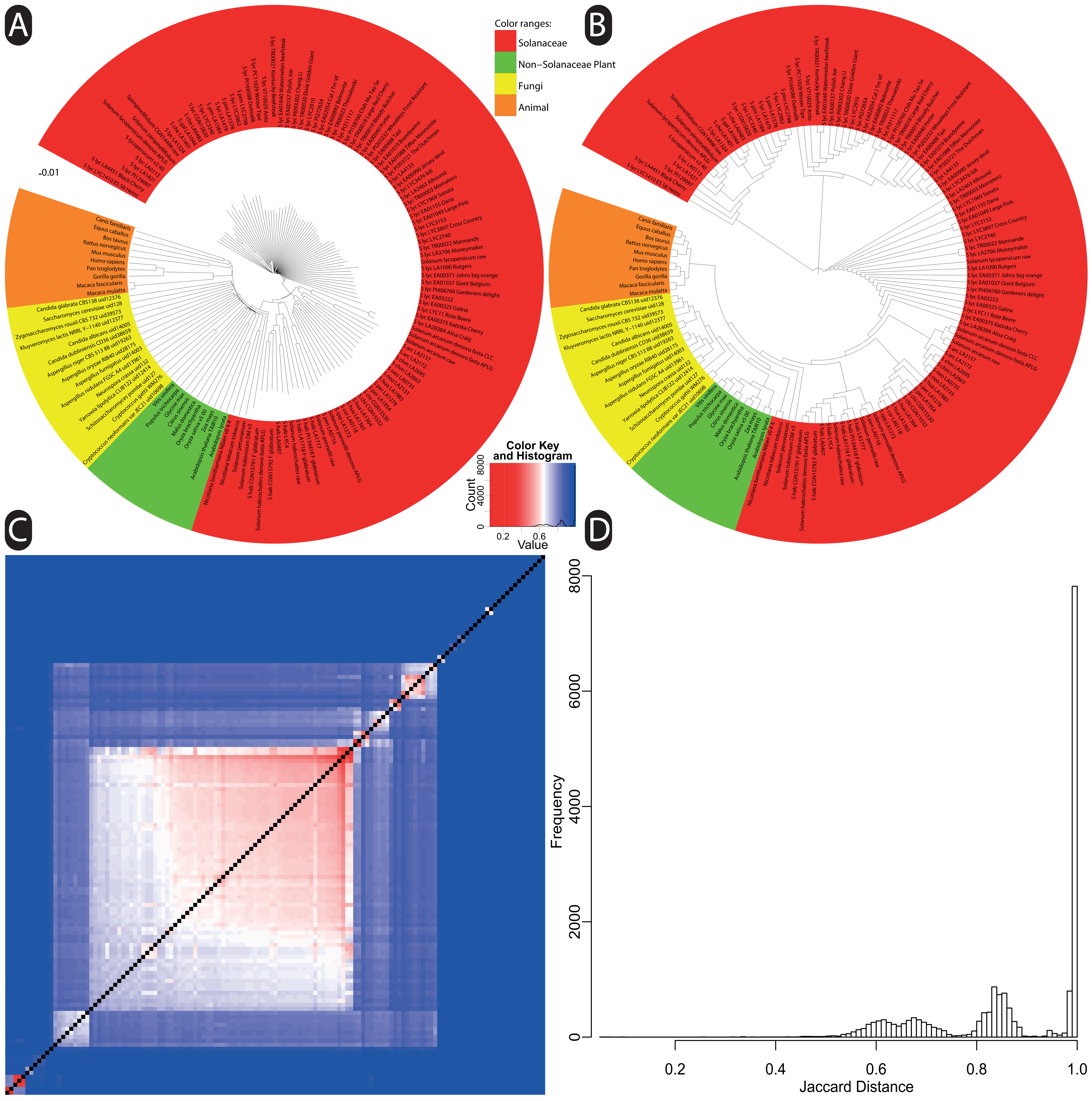

A
B
C
D
Color ranges:
Solanaceae
Non–Solanaceae Plant
Fungi
Animal
-0.01
Canis familiaris
Equus caballus
Bos taurus
Rattus norvegicus
Mus musculus
Homo sapiens
Pan troglodytes
Gorilla gorilla
Macaca fascicularis
Macaca mulatta
Color Key
and Histogram
Count
0
4000
8000
0.2
0.6
Value
Frequency
0
2000
4000
6000
8000
0.2
0.4
0.6
0.8
1.0
Jaccard Distance
